# Spur-winged lapwings show spatial behavioural types with different mobility and exploration between urban and rural individuals

**DOI:** 10.1098/rspb.2024.2471

**Published:** 2025-01-08

**Authors:** Michael Bar-Ziv, Hilla Ziv, Mookie Breuer, Eitam Arnon, Assaf Uzan, Orr Spiegel

**Affiliations:** ^1^ Faculty of Life Sciences, School of Zoology, Tel Aviv University, Tel Aviv 6997801, Israel

**Keywords:** biotelemetry, HIREC, movement ecology, exploration–exploitation trade-off, waders

## Abstract

Understanding how wildlife responds to the spread of human-dominated habitats is a major challenge in ecology. It is still poorly understood how urban areas affect wildlife space-use patterns and consistent intra-specific behavioural differences (i.e. behavioural types; BTs), which in turn shape various ecological processes. To address these questions, we investigated the movements of a common resident wader, the spur-winged lapwing (*Vanellus spinosus*), hypothesizing that urban individuals will be more mobile than rural ones. We used an ATLAS tracking system to track many (*n *= 135) individuals at a high resolution over several months each. We first established that daily movement indices show consistent differences among individuals, acting as spatial-BTs. Then focusing on the two main principle components of lapwings’ daily movements—mobility and position along the exploration–exploitation gradient—we investigated how these BTs are shaped by urbanization, season (nesting versus non-nesting) and sex. We found that urban lapwings were indeed more mobile in both seasons. Furthermore, urban females were less explorative than rural females, especially during the nesting season. These results highlight how urbanization affects wildlife behaviour, even of apparently urban-resilient avian residents. This underscores the need to consider possible behavioural consequences that are only apparent through advanced tracking methods.

## 1. Introduction

The global human population is steadily increasing, resulting in rapid urbanization and development [1]. These changes alter the availability and distribution of resources and threats, fragmenting habitats, and thus affecting ecological connectivity and habitat suitability [2]. Species may vary considerably in the direction and magnitude of their reactions to urbanization. Some may suffer from genetic isolation, fitness reduction and ultimately face (local) extinction [3]. On the other end of the spectrum, a small subset of species become ‘urban exploiters’ that benefit from these changes [4,5]. Nevertheless, despite the accumulating knowledge on behavioural responses to urbanization, there is still a lack of comprehensive synthesis on how it affects animal movement, and whether behavioural changes are repeatable. This gap limits our ability to predict the outcome of the ongoing urbanization for a species’ behaviour in different contexts.

A reduction in local movement of animals from more urban areas is common in mammals [6] but the direction of effect may differ among systems. A growing number of studies have shown that populations examined in urban settings tend to present unique sets of behaviours, such as bolder and faster exploration [7,8]. Some of these studies indicate that behavioural variation goes beyond simple plasticity and reflects consistent among-individual differences, also known as animal personalities or ‘behavioural types’ (BTs; [9,10]). Classical BTs include traits such as boldness, aggressiveness and exploration, and are typically studied by means of repeated behavioural assays in controlled settings [11,12]. Anthropogenic settings can non-randomly favour certain BTs that are better acclimated or adapted to such novel environments. For example, previous studies found that individuals are less responsive to an approaching threat [13,14]. Urban shrews, for instance, were found to be consistently bolder and more aggressive [15], and urban female lapwings (*Vanellus spinosus*) were calmer and more tolerant to threats than their rural ones [16]. Consistent behavioural changes, in turn, may ultimately lead to genetic adaptation, assortative mating and speciation [17,18]. Very few studies, however, have investigated how urban settings affect the spatial behaviours of their inhabitants, which can result in consistent movement-related differences [19].

Some BTs implicitly include or associate with movement (e.g. activity and exploration), and these aspects have been receiving growing attention recently. Such consistent differences in movement and spatial patterns are often referred to as spatial-BTs [20–22]. Spatial-BTs are typically inferred from tracking data *in situ*, thus while they offer many repeated measures in time (e.g. seasonal or daily movement patterns [20,23,24]), they lack the control setting used for characterizing more classical BTs. Differences among spatial-BTs can reflect intrinsic differences in the condition and personality (BTs) among individuals, often enhanced by the environments in which they reside. Indeed, spatial-BTs often show more pronounced differences than BTs: repeatability (*R*) is the common index used for quantifying individual consistency in behaviour (BTs and spatial-BTs), and it usually examines the differences between populations. Repeatability is bounded between *R* = 0 (non-distinguishable individuals) and *R* = 1 (individuals are fully behaviourally consistent and distinguishable [25]). A recent meta-analysis of 200 studies examining repeatability in movement found higher values for spatial-BTs (*R *~ 0.67 [21]) compared with the common mean across classical BT assays, estimated around *R* = 0.37 [26,27]. This higher value for spatial-BTs may represent the additional contribution that a heterogeneous environment has on movement patterns among individuals (i.e. individuals move differently both owing to their different BTs and their different environments).

Despite the high repeatability of spatial-BTs, intraspecific (or even intra-population) differences in movement may be challenging to identify, compared with the pronounced differences in movement patterns *among* species (e.g. migratory versus residents) or between different foraging strategies [28]. New tracking technologies that provide high-resolution data may help address this challenge by enhancing our ability to detect finer movement patterns that could otherwise go unnoticed, along with their associated spatial-BTs and their ecological consequences [21,29]. Examples demonstrating consistent differences in daily movement patterns, over and above the sex-related and seasonal differences, include, for instance, brown bears (*Ursus arctos*), which differ in maximum daily displacement and other movement indices (*R* = 0.16–0.61 [20] Similarly, burbot (*Lota lota*) have a repeatable home range, despite seasonal variation [30]. White ibis (*Eudocimus albus*) in Florida demonstrated individual specialization in recently urbanized areas [31]. This specialization has led to consistent age-related differences in their daily and seasonal movements, and as a result, reduced connectivity among habitats. This reduction in connectivity illustrates the potential interactive effect of internal factors (like individual specialization and age-related behaviour) and external factors (such as urbanization) in shaping spatial behavioural traits (spatial-BTs), which may result in significant ecological consequences [19,31]. More broadly, spatial-BTs and their composition in a population can fundamentally alter population-level processes such as dispersal, foraging and social interactions [32–34].

Here, we examined whether individuals from more urban environments demonstrate consistently different movement patterns compared to conspecifics nearby, dwelling in less urban areas. A recent meta-analysis, examining the effect of human disturbance on the movement patterns of multiple taxonomic classes has shown that urbanization strongly affects birds’ movement patterns [2], leading to a substantial increase in their home-range size and movement distances. Accordingly, we expect urban birds to exhibit higher mobility, flying greater distances than their nearby conspecifics. To this end, we captured and tracked resident spur-winged lapwings (*V. spinosus*), comparing individuals that were more ‘rural’ (i.e. living outside urban settings, mostly near water ponds or open fields) with others that were ‘urban’ (i.e. living mostly in settlement and built-up areas). Working within a restricted geographical area with both urban and rural habitats adjacent and well within reach, ensured that this variation reflected an individual’s preference rather than an inability to reach other habitats. First, we investigated whether the lapwings showed spatial-BTs in their movement. We hypothesized that individuals would consistently differ from each other, showing high repeatability in indices of daily movement, as was reported for various avian species showing spatial-BTs [21]. Then, turning to our main question, we asked whether the movement behaviours of urban individuals differ from those in rural areas, in addition to commonly reported internal factors (sex and weight) and external factors (season) shaping animal movement [35]. Having previously found that urban lapwings are bolder and exhibit unique correlations between behaviours that are not observed in their non-urban counterparts (*in situ* [14]; *ex situ* [16]), we expected urbanization to also affect their daily movement. Following Doherty *et al*. [2], we predicted that urban lapwings would demonstrate higher mobility and cover greater distances, while rural lapwings exhibit lower mobility, covering shorter distances. To test these predictions, we compared the movements and spatial-BTs between urban and rural residents using high-resolution tracking.

## 2. Material and methods

### (a) Study area and system

**Figure 1.**
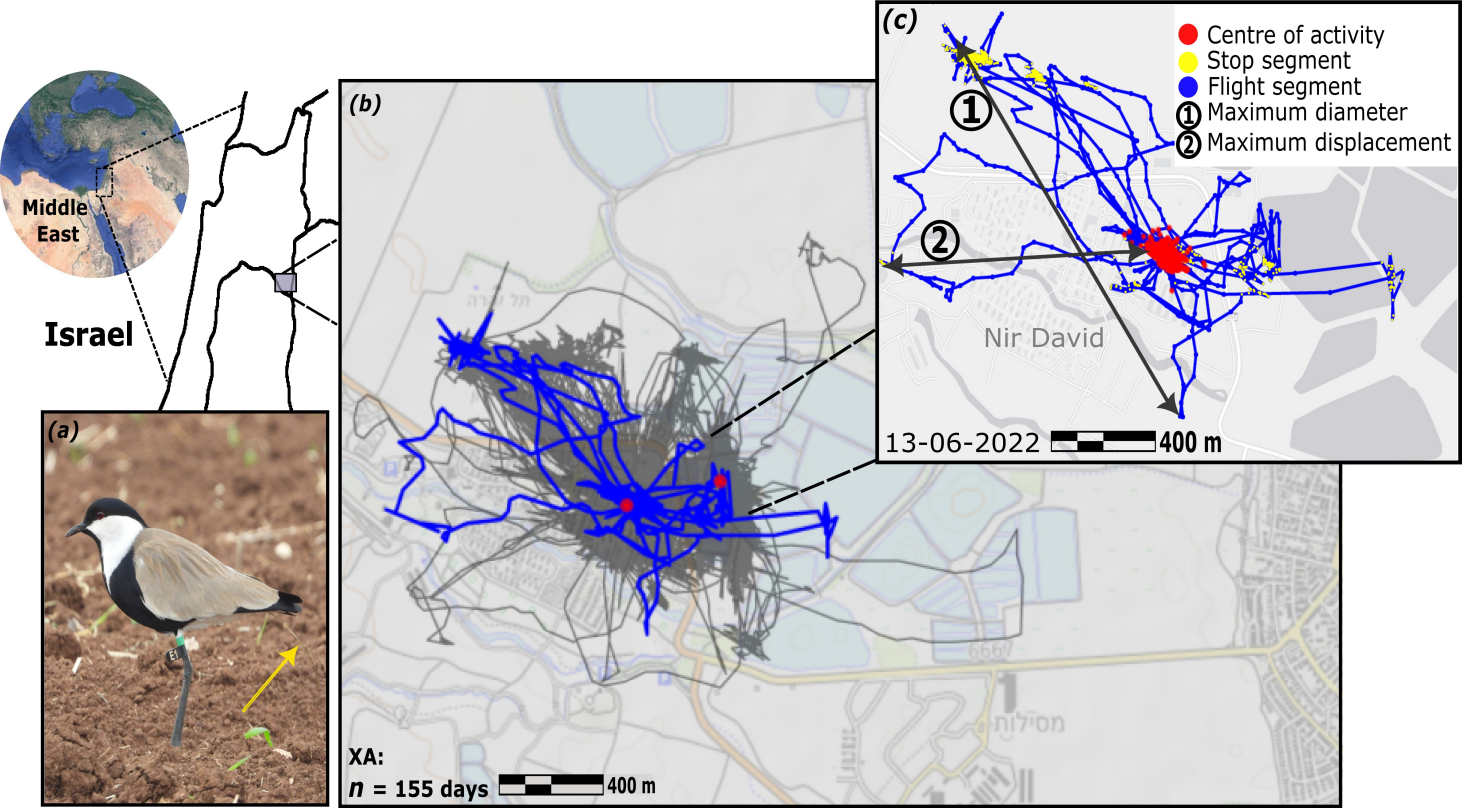
(*a*) Spur-winged lapwing (*Vanellus spinosus*), equipped with an ATLAS tag (yellow arrow indicates the antenna), and identification flag (E1; credit: Hilla Ziv). (*b*) A trajectory of an urban individual (XA, dwelling in Kibbutz Nir David), over 155 days (narrow grey lines), with one day (13 June 2022) highlighted as a blue line and enlarged in the inset. (*c*) The inset shows the segmentation and indices of this particular day. Yellow and red dots indicate ground stops and the centre of activity (areas where the lapwing stops and spends more than 75% of its overall tracking duration), respectively. Arrows indicate (1) the maximum daily displacement (max bee-line distance between any two locations within a day) and (2) the maximal distance from the centre of activity.

The spur-winged lapwing (*V. spinosus*, hereafter lapwing; figure 1*a*) is a common resident wader throughout the Middle East [36]. Lapwings are habitat generalists, thriving in a wide range of environmental conditions and open habitats, typically near water sources, fields, ponds and marshes. They also adapt well to urban and built areas and can often be found in parks and on lawns. Lapwings show bolder behaviours in urban settings [14] and their behavioural adaptations and habitat generality have enabled them to expand their distribution throughout Israel. This is in contrast to many other waders and ground-nesting birds whose populations are declining owing to growing human disturbance [37].

Nesting season lasts from March to August, with weak synchrony among pairs and repeated nesting attempts within a season if needed. During nesting, lapwings often form loose colonies and are very protective of their nest and chicks, mobbing animals (including humans) and attacking them with their spurs. Previous findings also suggest that lapwings display strong philopatry, regularly returning to the same nest locations and generally exhibiting limited dispersal to distant areas (Michael Bar-Ziv , Hilla Ziv & Orr Spiegel, 2023, unpublished data).

The present study was conducted in the Harod and Beit She’an valleys in plasticity can facilitate evolution in urban enviorth-east Israel (32°31'44.4" N, 35°26'30.1" E; figure 1), where the summer months are extremely hot (reaching up to approx. 40°C daily max) and winter is cooler ( approx. 20°C daily), with approximately 400–500 mm of annual precipitation. The valley is bounded by the Gilboa ridge to the southwest, shallow hills to the north and the Jordan River to the east. It includes a mosaic of habitats, mostly agricultural fields with approximately 10 small human settlements and one mid-size town (Beit She’an, approx. 20 000 inhabitants). There are also several natural national parks, many artificial fishponds (for agricultural growth) and other perennial water sources.

### (b) Data collection

#### (i) Captures and tagging

For movement tracking, we captured nesting lapwings during 2019–2022. We searched the study area for nests and placed a walk-in trap on them. Upon capture, each lapwing was banded and fitted with a standard flag for field identification (Interrex Ltd, Poland; https://colour-rings.eu/). Morphological measurements comprised body weight and the length of wing, tarsal and spur. When possible, sex was determined from spur length (males > 10 mm, females < 7 mm), and otherwise by means of genetic markers from a commercial provider (Karnieli Vet Ltd, Israel; https://www.karnieli-vet.com/), using feather samples collected from all individuals. Finally, because the lapwings at our study site weighed approximately 184 ± 11.4 g (x̄ ± s.d.; *n* = 129), we used 7 g tracking device tags (described in §2b(ii)) attached in a leg-loop configuration (<4% of body weight, including harness [38]). The birds were released back at the capture site (typically within 40 min), with the exception of 28 birds that were released after three weeks of behavioural trials in captivity [16]. A total of 211 transmitters were deployed on 194 individual adult lapwings (2020 = 68, 2021 = 74 and 2022 = 69), including 17 re-captures in subsequent years.

#### (ii) The tracking system

To obtain the fine-scale tracking data at the high frequency required to answer our questions on movement behaviour [29] we used Advanced Tracking and Localization of Animals in real-life Systems (ATLAS; [39,40]). This system is based on a reverse-global positioning system (GPS), in which the bird-borne tags emit a unique-ID signal at set intervals that is detected by an array of fixed base stations deployed at high vantage points in the region (tower-mounted antennas). Signals detected by three or more base stations enable localization of the tag from their differential times of arrival. The accuracy of the fixes is affected by the number and configuration of antennas participating in each localization, typically within a 3–10 m median accuracy [41]. The off-board calculations (rather than by the tag itself) make the tags cheap and energy-efficient compared with GPS tags of similar weight, thus providing high-frequency data over extended periods. In this study, the tags were set to be transmitted at 8 s intervals (1/8 Hz), with a practical lifetime of up to 315 days. The deployment of our particular ATLAS system comprised 19 base stations covering approximately 350 km^2^ (see [24,42] for details). Tracking started during the nesting season (March–August), and many of the trajectories extended into the non-nesting season (September–February), allowing us to include seasonal differences in the analyses, while addressing individual consistency in behaviour (i.e. spatial-BT), on an urbanization gradient.

### (c) Movement data analysis

#### (i) Movement indices

The movement data comprised a huge and noisy dataset. Before extracting movement indices and home-range estimates, we pre-processed localization following the pipeline described by Gupte *et al*. [43]. To ensure the quality of the daily track as well as the reliability of their urbanization level we excluded from further analyses days with ≤1500 localizations and individuals with less than 60 viable tracking days. All analyses were done in R language v. 4.3 and Rstudio [44]. The filtering and segmentation of the daily trajectories into flight segments intermitted with stops were executed with the packages ‘ToolsForAtlas’ [45] and ‘atlastools’ [46].

To determine whether lapwing movements present spatial-BTs, we characterized their daily movements using several movement indices [23]. The centre of activity (COA) was defined with a 100 × 100 m grid, as the area where the lapwings spent more than 75% of their total tracking time (figure 1*c*–*d*). For the vast majority of individuals (115 out of 135), the COA was a single location (of two or a few adjacent grid cells), while the remaining 20 individuals had their COA divided among 2–3 separate locations, at least >200 m from each other. Overall, we calculated the daily: (i) total time in COA (minutes; see the electronic supplementary material, table S1 for index definitions); (ii) maximum displacement from the COA (the distance to the furthest location in the daily trajectory, in metres); (iii) total flying distance (in metres); (iv) number of flying segments, (v) flying speed (m s^−1^); (vi) maximum diameter (the distance between the two most distant locations in a daily trajectory, in metres; see figure 1*c* for an example); (vii) daily locations visited; (viii) daily unique locations visited; (ix) re-visitation rate (total visited locations/unique locations visited on the map); and (x) total time on the ground.

### (d) Urbanization-level estimates

To determine whether the affinity to urbanization affects the lapwings’ movement behaviours, we estimated the coverage of urban habitat within their core home range (HR) similar to previous studies determining habitat-type percentages around nest locations or within a fixed-radius capture area [16,31,47]. Given lapwings’ strong philopatry, they are remarkably suitable for this method and tend to concentrate their activity within a confined range. As an index of their core HR (where they spent most of the time), we used the median kernel density estimation (KDE; of 50%). KDE considers the number of locations as well as their given density, which is represented by a grid size, and the median indicates expected usage [48]. For our analysis, we consider urban habitats as areas where people live, such as small villages or cities including lawns and roundabouts, or open areas within settlement borders. Rural habitats, in contrast, include fields and ponds with much lower human infrastructure (e.g. buildings) and activity (electronic supplementary material, figure S1). Rural lapwings readily alternate between fields and ponds (but less so with the urban area), justifying their consideration as a unified ‘rural’ category.

For determining the core HR (KDE 50%) we subsampled our data into 1 h intervals, thus reducing potential biases caused by auto-correlation of our high-resolution locations (a prerequisite for KDE) for this particular analysis. Given the biology of our species, which typically spends most of the time on the ground with short flight segments in between, for this analysis we considered the non-flight locations only. Finally, we combined habitat polygon layers of urban areas (‘National Topographic Database (BNTAL)’ [49]) and calculated the percentage of built-up in each lapwing’s core home range. This index (ranging from 0 to 100%) was used as a proxy for the bird’s level of urbanization, and individuals could readily be classified into ‘urban’ and ‘rural’ individuals. The index showed a clear bi-modal distribution with one peak below 30% (rural) and one above 60% (urban, electronic supplementary material, figure S2). One individual with an intermediate level was not included in further analysis. To validate the suitability of the 1 h interval as a representative measure, we analysed the distribution of urban environment percentages across 1, 2, 3 and 4 h intervals and found no significant differences in the core HR habitat usage (electronic supplementary material, figure S2). To ensure that observed differences in movements between the two groups (if any) are not an artefact from differences in the ATLAS position accuracy (built areas have more radio interference) we also compared the system reported localization error between groups and found no significant effect (electronic supplementary material, figure S3).

### (e) Statistical analysis

#### (i) Statistical modelling

After extracting the above-noted 10 daily movement indices, we calculated their repeatability and reduced the dimensionality of movement with a principal component analysis (PCA). For index repeatability, we have first transformed them to fit a normal distribution (electronic supplementary material, table S1), and then modelled each index with individual identity as a random effect and 1000 bootstraps for confidence interval estimation, using the package ‘rptR’ [27]. All indices that were significantly repeatable (*p* < 0.05) were included in the PCA, implemented with the ‘prcomp’ package from a base ‘stats’ package in R [50]. The first two principal components (PC1 and PC2) were used as the main dependent variables in subsequent models investigating the effect of urbanization on movement. In addition, we have calculated the repeatability of these two PCs and examined how PC’s repeatability changed between seasons and habitat types (urban versus rural).

To quantify the effect of different ecological predictors on each PC, we used a set of generalized linear mixed models (GLMMs), in a model comparison framework [51]. All models included bird ID as a random factor. Fixed effects varied among models and included: (i) our main predictor of interest—the urbanization level (urban or rural), as well as the additional predictors of: (ii) sex (male or female); (iii) season (nesting or non-nesting); and (iv) initial weight (scaled). Furthermore, because the date can have a strong effect on lapwing movement, we included a correlated autoregressive term in the model with a date for each ID. In the model set for comparison, we also included models with two- and three-way interactions of urbanization with the other three predictors because the effect of urbanization on movement can be mediated by these predictors. We implemented model selection with the ‘MuMIn’ package [52] and included only models with delta Corrected Akaike Information Criterion (AICc) ≤ 4 in the model averaging. Finally, after this analysis established an effect of the urban habitat on lapwings’ movement PCs, we explored the possible reason underlying this pattern by identifying specific movement indices that were associated with urbanization differences. To this end, we have tested with a two-way ANOVA the effects of urbanization, season, sex and their interactions on an individual’s mean index value for each of the nine indices. The *p*-values were adjusted to control for multiple comparisons with the Benjamini–Hochberg correction [53].

## 3. Results

A total of 135 lapwings met our inclusion threshold (64 males, 65 females and six unknown sex). Tracking duration was 194.7 ± 71.2 days (x̄ ± s.d.; range 60–315), reaching a total of 26 281 tracking days (spread over 1084 calendric dates). During the breeding season, the tracking durations were 75.4 ± 36.4 days, and 130 individuals were also tracked into the non-breeding season for 122.5 ± 65 days. Differences in tracking durations among individuals mostly reflect tag performance (pre-maturation failures), and in rare instances also individuals that left the study region during tracking (*n* = 8). Broadly speaking, the results show strong consistent individual differences in lapwing movements and an effect of urbanization status (21 urban and 114 rural individuals) on their movement, as detailed below.

### (a) Lapwings demonstrate spatial-behavioural types in their daily movements

**Figure 2.**
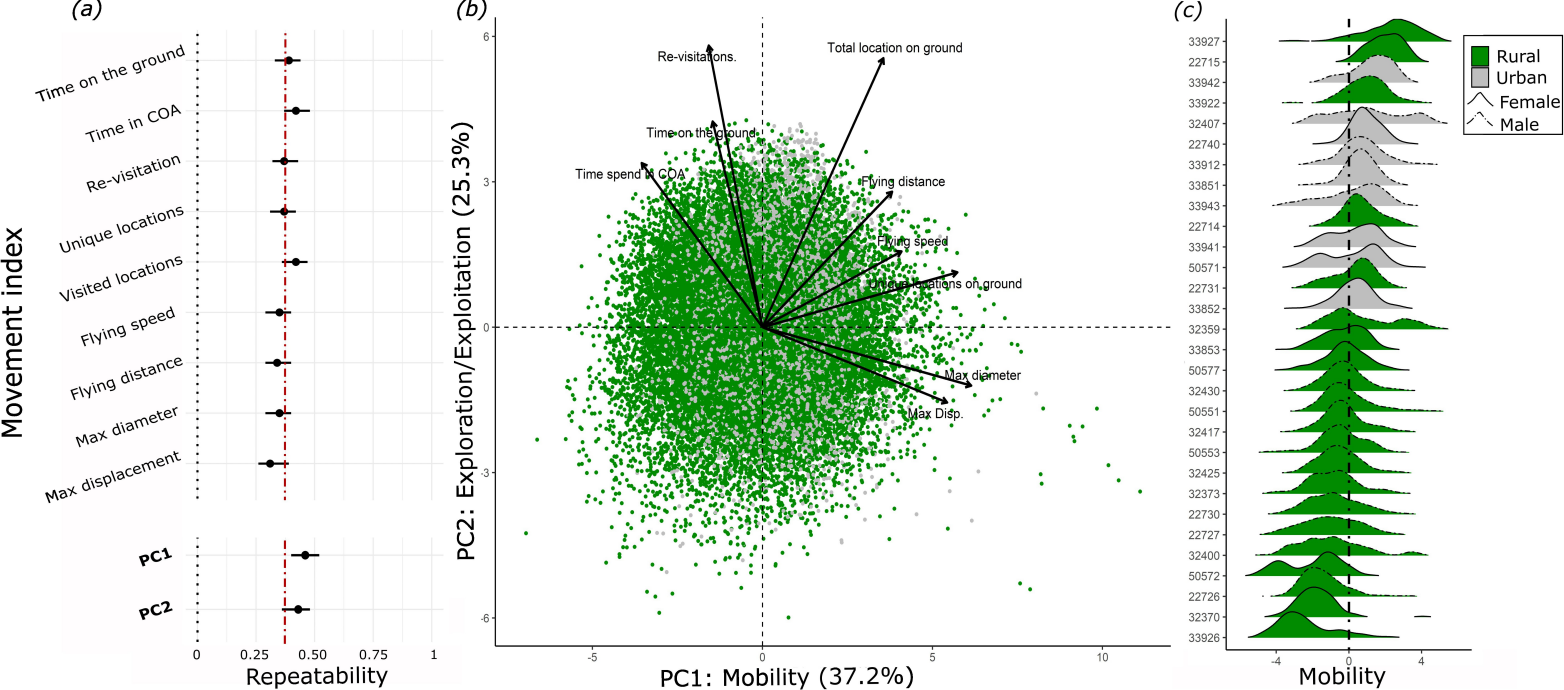
Repeatability and principal component analysis (PCA) of movement indices summarizing 26 281 tracking days of 135 lapwings. (*a*) Repeatability values and their 95% confidence intervals for daily movement indices are included in the PCA. Repeatability values for PC1 and PC2 are also presented at the bottom. The vertical red-dashed line indicates the *R* = 0.37 suggested as a mean of BT [26]. (*b*) A PCA bi-plot showing daily values and loadings for the nine indices of daily movements PC1 (37.2% stress): mobility and PC2 (25.3% stress): exploration–exploitation (ExE). Points indicate specific days (*n* > 25000), coloured by habitat. Higher PC values for the two axes indicate longer flight distances and a tendency to revisit the same location, respectively. (*c*) Density plots for 30 randomly selected individuals, demonstrating individual differences in their mobility (PC1). Colours indicate the bird’s habitat and line type represents their sex. Note that urban birds tended to show higher mobility.

To determine whether the lapwings’ movement behaviours can be considered as spatial-BTs, we established repeatability across the entire tracking period (i.e. both seasons combined) of our 10 movement indices, as well as for the two PCs from the PCA. Indeed, we found significant repeatability in all 10 indices (all *p* < 0.05; figure 2*a*; electronic supplementary material, table S1). Repeatability values ranged between 0.31 and 0.42, and most were close to the 0.37 value that was suggested as a typical R value in a large meta-analysis of BTs [26] but lower than that of spatial-BTs [21]. We have excluded the number of flying segments, which was strongly correlated (negatively) with total locations on the ground (*r*^2^ > 0.95), and included all nine other indices in the PCA (figure 2*a*).

The two PCs explained 37.2% and 25.3% of the total variance, within eigenvalues of 3.3 and 2.2, for PC1 and PC2, respectively (figure 2*b*). PC1 had high positive loading for indices related to the birds’ mobility (e.g. max displacement and flying speed) and negative loads for indices indicating more sedentary behaviours (e.g. time spent in the COA). Accordingly, PC1 can be interpreted as ‘mobility’, with days of higher values implying more movement. PC2 had positive loading for indices implying a tendency to revisit the same locations (namely re-visitation rate and time spent in the COA). Hence, we interpret this axis as an ‘exploration–exploitation’ (ExE) continuum , with a higher PC2 value indicating a tendency to exploit familiar places and low values indicating a tendency to more exploratory behaviour. Note that in contrast to a common (somewhat ambiguous) reference to exploration as being more mobile, here we distinguish between the two aspects: the range of movement (mobility) and the tendency to revisit the same locations (ExE).

Both PCs were strongly repeatable across days of the same individual (PC1: *R* = 0.46, CI = [0.4,0.52], *p* < 0.05; PC2: *R* = 0.43, CI = [0.36,0.48], *p* < 0.05; figure 2*a*; electronic supplementary material, table S1), and independent of each other, also at the aggregated individual level (*ρ* = −0.02, *p* = 0.76; electronic supplementary material, figure S5). Consequently, both axes can be considered as independent spatial-BTs, reflecting individual consistent movement patterns. Testing repeatability in different seasons revealed that for both mobility and ExE, the movements in each season were more repeatable than over the entire year combined. Rural lapwings exhibited higher repeatability in their mobility compared to urban lapwings, but lower repeatability in their ExE. This pattern remained consistent when analysing habitats and seasons separately, showing higher overall repeatability values while maintaining consistent differences between habitats (electronic supplementary material, figure S6, table S2).

### (b) The influence of urbanization on lapwing movement behaviours

After establishing that lapwing movement reflects two independent spatial-BTs (mobility and ExE), we analysed whether these were affected by an individual’s habitat, using the 129 individuals with known sex (total 25 243 days, urban = 19, rural = 110). To ensure that the GLMMs residual meets the normality prerequisite of the residuals, we also removed approximately 0.5% of the days that were identified as outliers in their PCA values (removing 145 and 77 observations (days) from PC1 and PC2, respectively). Working in a model comparison approach, we found that both PCs were affected by an individual’s urbanization status. For mobility, we found that urban lapwings generally moved more, and for the ExE, the interaction of urbanization with sex and season was important, as detailed below. To ensure that these effects were not a result of the sample size differences between habitats, we performed an additional validation establishing that the effect of urbanization remained similar also after sub-setting from the rural group and randomizing subsets for the model (electronic supplementary material, figure S4).

#### (i) Effect of urbanization on mobility

**Figure 3.**
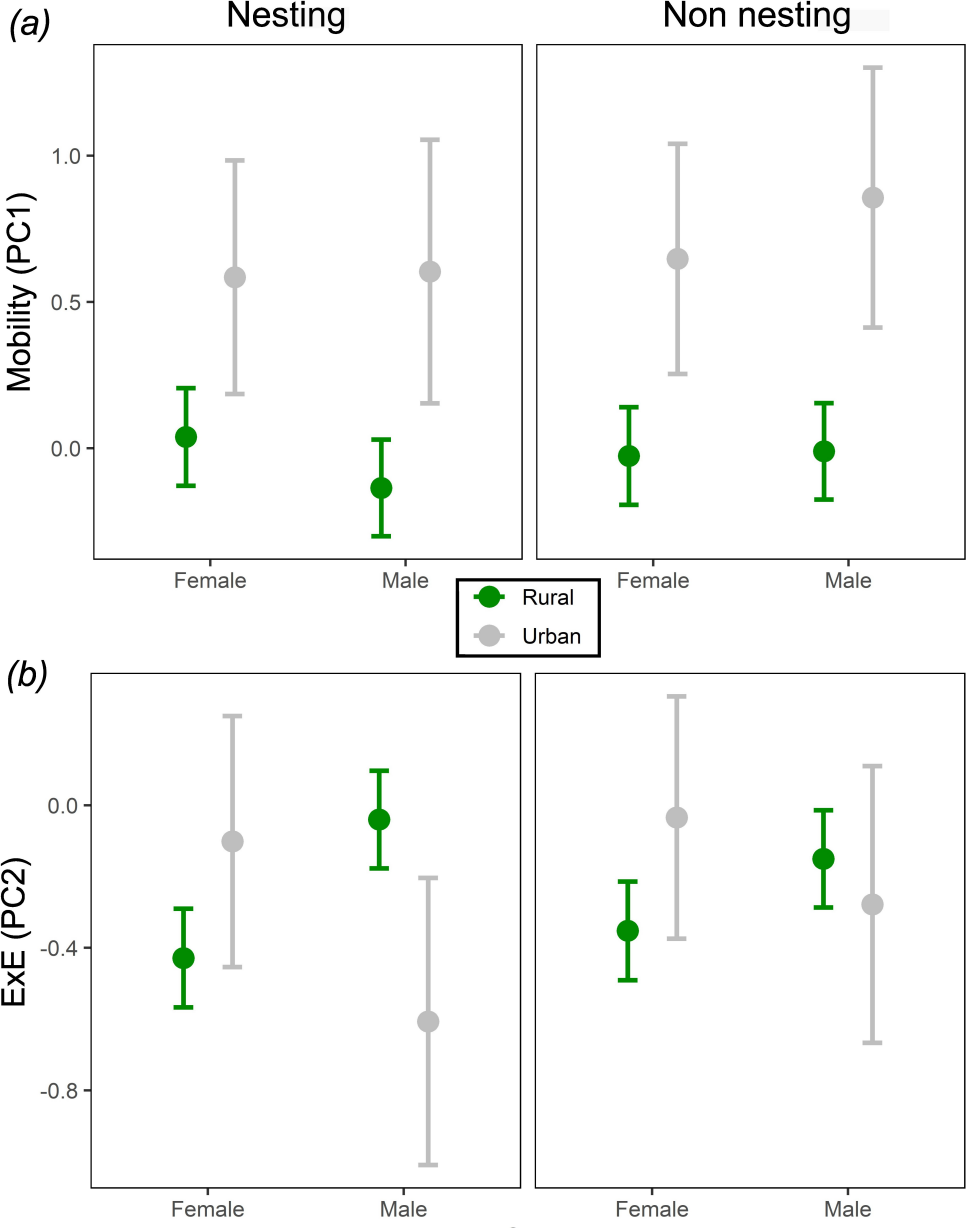
The results of the marginal average model for the effects of urbanization status on lapwing movement PCs, separated by season and sex. Colours indicate urbanization status (grey = urban, green = rural), dots represent the model mean marginal effect and error bars the standard error of model marginal effects across all tracking days. For (*a*) mobility (PC1), urban lapwings (grey) were more mobile than rural ones (green), in both seasons. For (*b*) ExE (PC2), the results indicate that differences were observed mainly during the nesting season, with urban females being more exploiters than rural ones, and males showing the opposite trend.

Urban lapwings were more mobile (figure 3*a*; electronic supplementary material, table S3a). Here, the top 33 models (differing in the presence of specific predictors) were included in the model averaging (cumulative weight of 68.5%), while all subsequent (147) models had ΔAICc > 4 (electronic supplementary material, appendix 2). All predictors were included in one or more of the top models. The predictors’ effect sizes implied that the urban lapwings were generally more mobile (0.62 ± 0.33; x̄ ± s.e. for all following estimates), but it is important to note that those results were marginally significant (*p* = 0.06). Sex did not affect mobility directly, but season was an important factor, and the interaction sex × season indicates males move more in the non-nesting season (0.1 ± 0.07). Yet, the models suggest that these differences are predicted to have a weaker effect on mobility compared with urbanization (figure 3*a*). We also found that mobility interacts with weight and sex, which is further elaborated in the electronic supplementary material, figure S7).

#### (ii) Effect of urbanization on exploration–exploitation

Similar to mobility, we found that urbanization status affected the ExE level of lapwings, but only when interacted with season and sex. The top 26 models were included in the model averaging (cumulative weight of 64.7%), and the remaining models all had ΔAICc > 4 (electronic supplementary material, appendix 3). Urbanization, sex, season and weight were all included in the top models together with their interactions. Interestingly, the interaction of sex and season implied that during the nesting season, urban females tended to be more exploiters (less exploratory) than rural females, while urban males tended to be slightly more exploratory than rural males (figure 3*b*; electronic supplementary material, table S3b). Overall, males tended to be more exploratory than females (0.36 ± 0.19), but when comparing between seasons males were less exploratory than females during the non-nesting season (−0.18 ± 0.07).

### (c) Movement-specific behavioural differences

**Figure 4.**
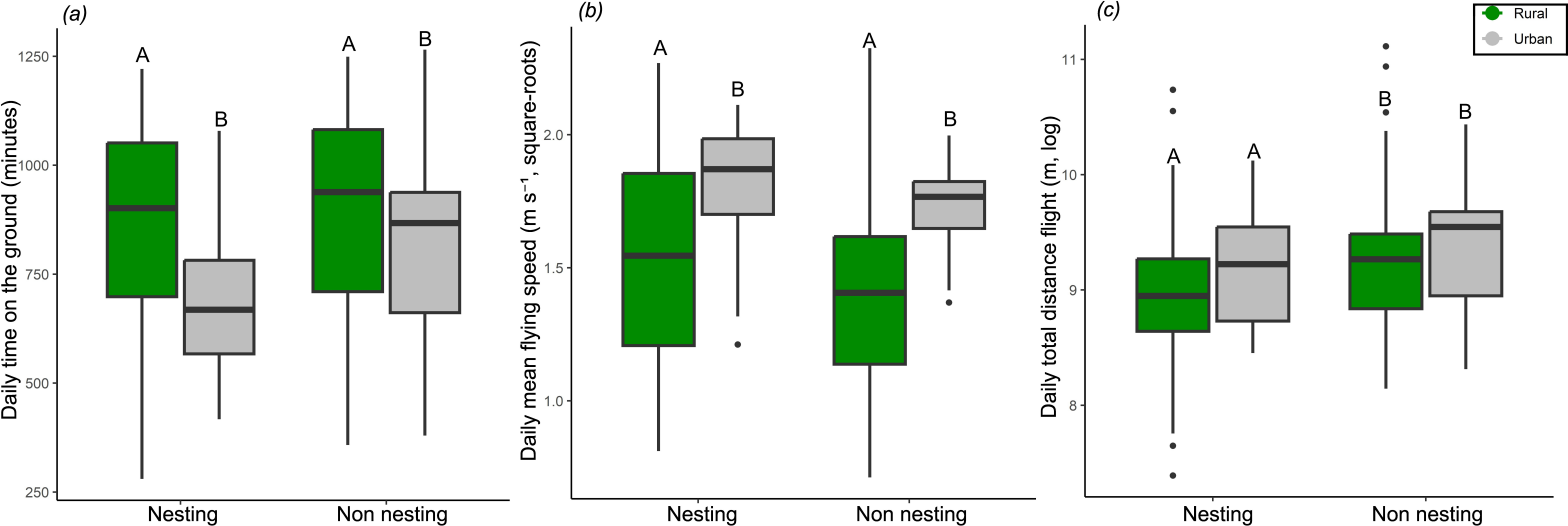
Behaviour-specific differences between rural and urban lapwings. Two of the nine behaviours composing the two PCs showed a significant effect of urbanization (*p* < 0.05, after adjusting with the Benjamini–Hochberg correction). In general, urban lapwings spend less time on the ground and fly faster (*a-b*). Lapwings also showed longer travel distances during the non-nesting season (*c*). Together these differences support the possibility that more frequent disturbances in urban habitats lead to the observed difference in mobility and ExE. Colours indicate urbanization status (grey = urban, green = rural), capital letters indicate statistically significant groups (electronic supplementary material, figure S8 for full comparison and multiple comparison correction). Note that the box plots present the distributions of mean individual values.

After establishing the difference in mobility (PC1) and ExE (PC2) between urban and rural lapwings, we turn to index-specific (*n* = 9) differences between habitats. Here, we found that two particular indices showed significant differences between groups, without any significant interaction with season or sex (full indices, electronic supplementary material, figure S7). First, urban lapwings tended to spend less time on the ground (*F*_1, 248_ = 10.6, *P-adj* = 0.007; figure 4*a*). Second, they also had faster flying speeds (*F*_1, 248_ = 20.24, *P-adj* < 0.001; figure 4*b*) compared to rural ones. These differences may be attributed to the urban habitat being more disturbed than rural environments, resulting in frequent flights and less time on the ground, as well as faster escape flight speeds. We also found a strong effect of season, independent of habitat origin, with longer travel distance during the non-nesting season (*F*_1, 248_ = 17.81, *P-adj* < 0.001; figure 4c). Possibly, these shorter distances during nesting resulted from the need to remain in the nest proximity during this season. Finally, while we tested for differences between sexes, there was no strong evidence supporting a significant effect in any of the specific indices (unlike the situation for mobility and ExE), and it was subsequently excluded from the index-specific comparisons.

## 4. Discussion

Our primary objective was to investigate the effects of dwelling in human-dominated environments (urban areas) on the movement and on the consistent differences among spatial-BTs of birds. To address this question, we focused on a common resident wader, the spur-winged lapwing, and tracked a large number of individuals at a very high resolution (approx. every 8 s) for relatively long periods (approx. six months). This extensive dataset was instrumental in revealing nuanced aspects of their movement behaviours, which would have probably remained elusive with conventional, low-resolution tracking methods [29]. Through a multifaceted study of their daily movement patterns (e.g. indices such as re-visitation rate, unique location visited and maximal displacement), we were able to identify two independent (PC) axes of movement that were both repeatable spatial-BTs: mobility (the scale of movement—namely, daily distance and flight duration) and ExE tendency (the shape of movement, patterns of re-visitation). Lapwings were classified as urban or rural (based on a bi-modal distribution of the proportion of built-up areas in their core home ranges). Models of their movement revealed a clear pattern of increasing mobility by urban individuals. Furthermore, during the nesting season, urban females were less exploratory than rural ones, while males showed the opposite trend. Further analyses showed that these differences were mostly owing to the urban lapwings spending less time on the ground as well as flying faster than their rural counterparts, possibly owing to higher disturbance rates in urban habitats.

To the best of our knowledge, the current study is among the first to show that both movement and the consistent intra-population differences in it (i.e. spatial-BTs) are shaped by urbanization. This (potentially very broad) effect of urbanization on space use and behaviour may drive substantial changes in subsequent ecological processes. In §4a–c, we first discuss the general implications of modified movements in human-dominated areas, and then the general importance of spatial-BTs in wild populations and urban settings. We conclude by acknowledging a few limitations of the current study and suggest future directions for addressing these topics.

### (a) The effect of urbanization on movement behaviour

Here, we observed that urban lapwings become more mobile. Although this pattern could be attributed to the dynamic nature of urban landscapes, with food availability and vegetation continuously changing [54], this seems an unlikely explanation in our case. Alternatively, the increased mobility of urban lapwings could result from frequent escape behaviours owing to the disturbance in these settings. This explanation agrees with the results of faster flight speed and less time spent on the ground by urban lapwing—both possible responses to frequent casual (often non-intentional) disturbance by passing humans or cars, pets and alike. Interestingly, urban lapwings assayed for their flight initiation distance at this area displayed bolder behaviours, initiating escape from shorter distances and to a shorter range [14]. Presumably, this should have resulted in lower mobility, not higher as reported here. The elevated mobility observed nonetheless (driven by faster flights and less time on the ground), may thus suggest that the disturbance rate is high enough to trigger more escapes and override the habituation and boldness effects [55]. Direct identification of the specific reason driving elevated mobility in urban settings (which may differ among species and study systems) may require an experimental approach or better quantification of the distribution of resources and threats in the different habitats that are not currently available for this system. Yet, regardless of the specific mechanisms, the increased mobility can impose some ecological costs for ground-feeding lapwings, as well as for many other species. Faster flights and less time on the ground usually result in higher energy expenditure, higher exposure to areal predators and shorter foraging opportunities (excluding areal insectivore species) highlighting some of the potential understudied costs of urbanization [56].

In addition to increased mobility, we found seasonal-, sex- and urban-dependent variation in ExE. First, during the nesting season, lapwings are less explorative, presumably reflecting the limitation imposed on their movement by the nest and chick during this season. This seasonal effect is more pronounced for rural birds, whereas urban individuals show lower seasonal variation in ExE. Second, rural females are more explorative than rural males in both seasons, which agrees with the accepted notion that avian females tend to be more mobile (e.g. [57]). While most studies to date probably did not have access to a suitable resolution method for measuring ExE *per se*, sex-dependent local movements were commonly documented. For instance, Adélie penguin females were shown to forage in more remote locations than males [58]. Third, urban females displayed less explorative behaviours than rural ones, mostly during nesting season. Similar to lapwings, the urban brushtail possum (*Trichosurus vulpecula*) also presented an interaction with sex and exploration [59], indicating that the effect of urbanization on both mobility and ExE merits further attention.

Several studies provide empirical support for the effects of urbanization on local wildlife movements that correspond with our findings. For example, urban coyotes show unique movement behaviours, which differ from rural ones, spending more time foraging and less time exploring [60]. In contrast to our finding of an increase in mobility in urban settings, a global comparison by Tucker *et al*. [6] indicated that non-volant mammals often reduce their movement in urban environments, primarily owing to physical barriers. Volant birds, are typically less restricted by barriers, and thus might show a different response to urbanization, as was indeed reported for home-range size comparison [2]. Yet, the differences in the response of birds to urbanization can be affected by the nuanced differences among species, study sites and methodology. Some species may even show a reduction in movement with higher human impact. For instance, Da Silveira *et al*. [61] found that the average flying speed of thrushes was lower in urban environments, not faster like in our case. The variation observed across studies in animal movements in response to urbanization highlights how species may experience the landscape differently, depending on factors such as foraging strategies or nesting behaviours. These diverse responses emphasize the need to study changes in behaviour in urban areas in the context of consistent individual differences.

### (b) Movement behaviours as spatial-behavioural types

An increasing body of literature acknowledges the ecological significance of consistent behavioural variation within populations [26,62]. More recently, this perspective has been embraced in the field of movement ecology, exploring the ecological consequences of individuality in movement [20,21,24]. The importance of these consistent spatial-BTs has been well established for key ecological processes like foraging, social network structure and ultimately for the population’s ability to adapt to changing environmental conditions [23,34,59]. Yet, a general challenge of almost any study inferring spatial-BTs and consistency from *in situ* tracking data—is that of the interpretation of consistency. Because the studied individuals are active in a heterogeneous environment, they might be repeatedly experiencing different conditions (if they are not nomads), which affect their behaviour. Thus the observed consistent behavioural differences may emerge even if they follow the same behavioural reaction norm [63]. This highlights the importance of partitioning variation in movement, and quantifying consistent among-individual differences.

Repeatability is the key index for assessing the strength of individuality of a given trait and is often considered as an upper limit for its heritability under certain conditions [27,64]. Here we found that our nine indices of movement exhibited a moderate degree of repeatability across indices (from 0.31 to 0.42). Reducing the dimensionality of movement with a PCA revealed two PCs with even higher values of individual repeatability—0.46 and 0.43 for PC1 and PC2, respectively. These values are on the high end of the mean repeatability value reported by Bell *et al*. [26] for BTs in general (approx. 0.37) and agree with their notion that BTs assayed under field conditions tend to show higher repeatability values. In a recent meta-analysis of spatial-BTs, Stuber *et al*. [21] found even higher values (0.59 to 0.8) than those reported here and provided several (non-mutually exclusive) explanations for these higher repeatability estimates for spatial-BTs compared with general BTs.

We suggest that our moderate spatial-BT values reflect the long tracking periods and the high-resolution tracking. Our dataset, with an average tracking period of over six months, incorporates clear changes in individual behaviour across seasonal conditions and the breeding cycle. Indeed, in agreement with the notion that lower repeatability estimates are expected over a longer period owing to lower temporal correlation in external factors [26] when testing for repeatability in different seasons, we found that it increases both movement behaviours (from approx.0.46 to 0.56 for season-specific repeatability). This effect was even more pronounced when separating the data by season and habitat, with values rising to nearly 0.64 in some cases. Furthermore, most of the studies and spatial-BTs in Stuber *et al*. [21] represent broader scale space-use patterns, such as home-range size and habitat preference, obtained at a coarser resolution compared with our daily analysis scale. While there is a growing body of literature that uses high-resolution data at comparable rates of seconds [29], these have usually been restricted to short observation periods, primarily owing to technological limitations and battery lifetime (e.g. [40,65]). Similar repeatability values of R < 0.4 have been reported for spatial-BTs of barn owls tracked at a similar resolution [24]. These findings highlight a potential interdependency between repeatability estimates, tracking duration and sampling resolution. Given the central importance of repeatability estimates for many ecological and evolutionary processes [25,27], future studies can directly explore these links by combining simulation studies with subsampling practices of empirical tracking datasets.

Finally, while the observed differences in spatial-BT between urban and rural individuals are prominent in our system, as well as a few other rare examples, such as brushtail possum [59], it remains challenging to determine precisely how these differences emerge. The relative contributions of human disturbance, non-random arrival of certain spatial-BTs to urban sites or behavioural plasticity and habituation by urban individuals [55] can all lead to the same patterns of urban-dependent spatial-BTs. More broadly, in order to fully understand the mechanisms and the ecology affecting spatial-BTs, it would be beneficial to combine tracking with experiments such as translocation (to determine whether behaviour remains consistent despite experiencing a different environment) or repeated BT-assays in a controlled environment, thus coupling spatial-BTs with their underlying BTs [20,23,66,67]. Additional studies incorporating these approaches will enable a more mechanistic insight into the effect of urbanization on animal movement.

### (c) Study limitations and concluding remarks

Despite our strong findings of robust spatial-BTs that are affected by urbanization and the accuracy of our system some limitations need to be addressed. First, several indirect effects of urbanization can contribute to the apparent difference. Factors like age, body condition, disease, parasites and social interactions can strongly impact bird movement, above and beyond the effect of the habitat itself [68]. Similarly, the nesting season may shift or shorten with increasing urbanization (e.g. in white ibis [31]), thus affecting urban movement indirectly. Such possible shifts in nesting timing also may contribute to the observed difference given our comparison using fixed nesting season, and future studies can account for such effects by nest monitoring *in situ*, and by monitoring additional predictors like parasite loads. Second, future studies could delve into variations within specific activity times within the day (such as night versus day activity window), or in other movement indices not covered here. Third, working within a confined region limits the range of urbanization gradient we cover. Neither really pristine habitats (with low human footprint), nor very urbanized ones (major cities with very high human footprint) are available within our area of coverage (the Harod and Beit She’an valleys). Instead, our gradient ranges from agricultural areas to small town and rural settlements. Lapwings do occupy suitable habitats within large cities [16]*,* and the effects of urbanization reported here may vary in magnitude or direction if evaluated across the full range encompassed by the species’ realized niches.

In summary, our study, employing high-resolution tracking, has revealed that lapwings in urbanized areas adapt their movement. We identified robust spatial BTs across various indices and were able to separate between lapwing’s mobility and ExE behaviours, two aspects of movement that are often not well distinguished. These two independent spatial-BTs were affected by interactions of the phenotypes (sex) with environmental factors (urbanization) and external elements (season). The ability to discern these interactions by means of movement underscores the significance of high-resolution tracking for revealing behaviours that otherwise are not possible to uncover. Our data indicate that individuals tend to occupy either urban or natural areas but not intermediate ones. If these behaviours are reinforced by non-random mating, future generations of these urban populations may undergo divergence from their conspecifics in nature [18]. In a world rapidly evolving towards increased urbanization, understanding how animals adapt their behaviours and use of space is crucial for mitigating the maladaptive consequences of the ongoing urban development.

## Data Availability

Electronic supplementary material is available online [69]. The codes, including filtering methods and metadata, are available on Dryad at [70]. The datasets generated and analysed during the current study are available in the Movebank Data Repository, https://doi.org/10.5441/001/1.629 [71]. Supplementary material is available online [72].
